# Burnout and quality of life in nursing staff during the COVID-19 pandemic

**DOI:** 10.1186/s12912-022-01168-7

**Published:** 2023-01-12

**Authors:** Silmara Meneguin, Isabelle Ignácio, Camila Fernandes Pollo, Heitor Marques Honório, Mayara Salles Gasparini Patini, Cesar de Oliveira

**Affiliations:** 1 Department of Nursing, Botucatu Medical School, Paulista State University, São Paulo, Brazil; 2 University of São Paulo, São Paulo, Brazil; 3 Department of Epidemiology & Public health, University College London, London, UK

**Keywords:** COVID-19, Burnout, Quality of life, Nursing, Coronavirus

## Abstract

**Objective:**

To investigate the association between burnout and quality of life among nursing staff at intensive care units during the new coronavirus pandemic and identify the influence of sociodemographic variables.

**Methods:**

A cross-sectional study was conducted with 109 nursing staff members at intensive care units of a public hospital in Brazil. Data collection involved the administration of a sociodemographic questionnaire as well as the WHOQOL-Bref and Oldenburg Burnout Inventory*.*

**Results:**

The participants presented a high state of burnout and a low score in the physical domain of the quality-of-life instrument. Men (*p* = 0.037), income (*p* = 0.011) and burnout (*p* < 0.001) independently influenced quality-of-life (*p* < 0.01). Age, being a nursing technician and working at two hospitals exerted an influence on burnout status (*p* < 0.05). A negative association was found between quality of life and burnout (*p* < 0.01).

**Conclusion:**

Our findings showed that burnout due to occupational circumstances affected negatively the quality of life perceptions of nursing staff working at intensive care units during the COVID-19 pandemic.

## Introduction

The pandemic of the new coronavirus (COVID-19) caused a substantial impact on the practice of nursing staff who work in intensive care units (ICUs) [1, 2]. During this period, these healthcare providers faced daily situations of exposure to the virus, fear of acquiring the disease, high numbers of deaths due to COVID-19 and long working hours, which exacerbated the level of work-related stress [3]. This unprecedented situation in the lives of healthcare providers required adapting to a new reality.

Over time, this context can contribute to mental and physical exhaustion, triggering burnout syndrome [4], which results from repetitive emotional pressure and is characterized by exhaustion, depersonalization and a diminished sense of personal achievement [5, 6].

Burnout syndrome is a multidimensional concept comprising three interlinked but independent dimensions i.e. emotional exhaustion, when workers demonstrate a lack of energy/enthusiasm for the practice of the service; disengagement, when workers become emotionally insensitive; and low sense of professional achievement, which is intrinsically related to the feeling of dissatisfaction with work [7–10]. The consequences of this syndrome affect the individual, health establishments and patients [6].

Among nurses and physicians who work in ICUs, the incidence of burnout syndrome is estimated to be 25 to 80%, with the degree of impairment ranging from mild to severe [6]. A recent study investigated burnout syndrome in healthcare providers who work in ICUs in Italy and concluded that 60% presented the syndrome during the period of the study. The authors also found that nurses had significantly higher degrees of anxiety and were more likely to have insomnia compared to other healthcare providers [11].

The word *burnout* is used to denote a state of energy depletion associated with intense frustration with one’s work situation [10]. After the publication of a study in 1974, this term was used to refer to a chronic condition characterized by stress, the depletion of resources and fatigue [12].

This type of occupational stress not only affects one’s work, but also other areas due to the interconnectedness of the aspects of one’s life [13]. Thus, individuals run the risk of developing burnout syndrome at work and also having negative impacts that compromise overall quality of life [14].

The World Health Organization (WHO) defines quality of life (QoL) as the perceptions individuals have regarding their position in life in the context of the culture and value system in which they live and with respect to their goals, expectations, standards and concerns [15]. However, the assessment of QoL, which is a subjective, abstract construct, is recognized to be a complex task. The WHO definition is profound and regards positive and negative facets of life in a multidimensional concept that addresses relationships between the environment and physio-psychological aspects, level of independence, social relations and personal beliefs [16].

A study recently conducted in India with healthcare workers who provided direct care for patients infected with COVID-19 found that 87% experienced a substantial reduction in QoL [17]. The literature also shows that COVID-19 has contributed to a greater occurrence of anxiety and sleep disorders, which not only exert a negative impact on the QoL of healthcare providers, but can also compromise their problem-solving skills [18].

There are several reliable and validated instruments to assess burnout and QoL such as the WHOQOL-Bref [19], Oldenburg Burnout Inventory [20], Burnout Assessment Tool [21], Maslach Burnout Inventory [22], Copenhagen Burnout Inventory [23] and Pines’ Burnout Measure [24].

The Oldenburg Burnout Inventory (OLBI) was developed to measure work related burnout. The OLBI scale does not only assess occupational factors and it can be applied to any occupational category [20, 25]. This instrument has been translated and validated in Brazil and does not require a license for its use i.e., it is free [20].

The WHOQOL-BREF, an instrument used to assess quality of life, is generic and multidimensional. It measures various aspects such as social integration, physical security, mobility, and body image and it can be applied not only to patients but also healthy individuals [19].

Although QoL and burnout among nursing staff have been widely explored in the literature during the pandemic, the present study conducted at a public hospital in the state of São Paulo, Brazil, is relevant, as the hospital is a regional reference institution for the treatment of COVID-19 cases. Thus, this investigation could contribute to the adoption of more comprehensive strategies for the prevention of burnout faced during pandemics in the hospital setting as well as the reorganization of care.

The following were the guiding questions of this study: How does burnout influence the QoL of nursing staff working in ICUs during the pandemic? Are sociodemographic and occupational variables associated with QoL and burnout in the context of the pandemic?

Therefore, the aim of the present study was to investigate the association between burnout and quality of life among nursing staff at intensive care units during the COVID-19 pandemic and identify the influence of sociodemographic and occupational variables.

## Methods

### Study design

An exploratory cross-sectional study with a quantitative approach was developed in the period between July 2020 and March 2021.

### Setting

This study was conducted at adult, neonatal and paediatric intensive care units of a public hospital located in the state of São Paulo, Brazil, which is a regional reference institution for cases of COVID-19.

### Population

Male and female nursing staff aged 18 or older who worked in ICUs providing care for patients infected by COVID-19 and who provided written consent to participate were included in the study. Those on leave during the data collection period, those who exercised administrative activities and those who reported not being emotionally fit to participate in the interview were excluded from the study.

### Calculation of sample size

Considering the prevalence of burnout among nursing staff to be 46%, a 95% confidence level and 10% margin of error, the minimum sample size was determined to be 94 participants. The following formula has been used:

$$n={\left(\frac{z_{\alpha /2}\sqrt{p\left(1-p\right)}}{\varepsilon}\right)}^2,$$

Where:

z_α/2 =_ 95% quantile of the normal distribution (1.96).

p = stress prevalence (46%) [26].

ε = margin of error (10%).

### Outcome measures

Three instruments were used for the data collection: a questionnaire addressing sociodemographic characteristics, the WHOQOL-Bref [19] and the version of the Oldenburg Burnout Inventory [20], both cross-culturally adapted for use in Brazil.

The WHOQOL-Bref is a self-complete instrument that contains 26 questions. Two questions on general quality of life (items 1 and 2) and the remaining questions are divided into four domains: physical, psychological, personal relations and environment. The WHOQOL-Bref is a 5-point Likert scale in which a higher score means a better quality of life. Each domain total score may range from 4 to 20, with higher scores meaning a higher quality of life [19].

The Cronbach-alpha value for the validated Portuguese version of the WHOQOL-Bref [19] was 0.91. WHOQOL-Bref values <60 indicate lower quality of life [27, 28]. In the present study, the translated and validated Portuguese version of the scale was used.

The OLBI is a Likert scale with 15 items divided into two domains: disengagement from work and exhaustion [20]. The disengagement from work aspect refers to the development of negative and cynical attitudes and behaviour at work. The exhaustion aspect refers to feelings of physical fatigue, need to have a rest, feelings of being overworked and emptiness towards work [25].

For the Oldenburg Burnout Inventory [20] a reliability value higher than 0.7 is considered ideal. The exhaustion dimension showed a reliability of 0.92 and the disengagement from work a reliability value of 0.88. This way, both dimensions showed high reliability [20]. According to Peterson et al., mean scores of ≥2.25 and ≥ 2.1 in the exhaustion and disengagement domains, respectively, are considered high [29].

In the present study, the Cronbach-alpha values for the WHOQOL-Bref and the Oldenburg Burnout Inventory were 0.67 and 0.76, respectively.

### Data collection and analysis

Each participant answered print versions of the questionnaires individually. The participants were assured anonymity in their answers and that refusal to participate would not have any negative consequences.

All variables were analyzed descriptively. The Shapiro-Wilk was used to check the normality of the data. Generalized linear regression models were run to determine differences in burnout and QoL scores according to sociodemographic and occupational variables (stepwise model). All analyses were performed using the IBM SPSS program, version 22. The level of significance was set at 5% (*p* < 0.05).

### Ethical aspects

This study received approval from the Human Research Ethics Committee of the Botucatu School of Medicine - UNESP (protocol number: 4.069.300) and was conducted following the guidelines for Strengthening the Reporting of *Observational* Studies in Epidemiology (STROBE statement) [30].

## Results

One hundred twenty-five nursing staff members met the inclusion criteria, 16 of whom declined to participate in the study, resulting in a sample of 100 and nine participants.

Table 1 displays the sociodemographic and occupational characteristics of the participants. The predominant characteristics were the female sex (92.6%), age between 31 and 40 years (39.5%), having a spouse/partner (67.9%), not having children (61.4%), a complete higher education (52.2%) and the Catholic religion (54.1%). The majority worked as a nursing technician (67.9%) in only one hospital (89.9%) during the day shift (62.3%) with a weakly workload of 40 to 49 hours (64.2%) and had not been placed on leave due to infection or a suspicion of infection by COVID-19 (65.1%).

**Table 1:** Sociodemographic and occupational characteristics of the participants. Botucatu, SP, Brazil, 2022

Variable	n	(%)
**Sex**		
Female	101	(92.6)
Male	8	(7.4)
**Age**		
Mean (±SD)	36	(±9)
**Marital status**		
With spouse/partner	74	(67.9)
Without spouse/partner	35	(32.1)
**Having children**		
Yes	42	(38.6)
No	67	(61.4)
**Educational level**		
High school	57	(47.8)
Higher education	52	(52,2)
**Occupation**		
Nursing technician	74	(67.9)
Nurse	35	(32.1)
**Work shift**		
Day	68	(62.3)
Night	41	(37.7)
**Number of jobs (number of hospitals)**		
One	98	(89.9)
Two	11	(10.1)
**Having been on leave due to COVID-19**		
No	71	(65.1)
Yes	38	(34.9)
**Religion**		
Catholic	59	(54.1)
Non-Catholic	50	(45.9)
**Weekly workload**		
30 to 39 hours	8	(7.3)
40 to 49 hours	70	(64.2)
50 to 59 hours	4	(3.7)
More than 60 hours	27	(24.8)
**Monthly family income (Brazilian currency)**		
≤ R$1000	1	(0.9)
R$1001 to 3000	42	(38.5)
R$3001 to 5000	40	(36.7)
> R$5000	26	(23.9)

In Brazil, nursing staff are categorised into three types. These are referred to as nurse, nursing technician, and nursing assistant [31]. A nurse is a highly qualified professional with technical and scientific competence who are responsible for the entire nursing staff. A nurse technician is a professional with technical training. They provide direct care to patients at clinics, hospitals, private households and at urgency and emergency services. A nursing assistant is a professional who does repetitive tasks such as hygiene care and comforting patients. They do not work in intensive care units [32].

Table 2 displays the median and interquartile range (25th to 75th percentile) of the factors on the Oldenburg Burnout Inventory and QoL domains. Regarding burnout, a higher median score was found for exhaustion (3.13) than disengagement (2.43), but both scores indicated a high degree of burnout. The most compromised aspects of QoL were the physical domain (12.6) and social relationships domain (13.5).

**Table 2:** Distribution of median (25th-75th percentile) of Oldenburg Burnout Inventory factors and WHOQOL-Bref domains. Botucatu, SP, Brazil, 2022

Burnout	Median	25th-75th percentile
**Factors**		
Exhaustion	3.13	2.75 – 3.63
Disengagement	2.43	2.00 – 2.71
**Quality of life**		
**Domains**		
Physical	12.6	11.4 – 13.7
Psychological	14.0	12.7 – 15.3
Environment	16.0	13.3 – 16.0
Social relationships	13.5	12.0 – 15.0

Table 3 displays the results of the multivariate analyses using generalized linear models for quality of life and burnout. Age (*p* = 0.022), burnout (*p* < 0.001), the male sex (*p* = 0.037) and monthly family income (*p* < 0.01) exerted a significant influence on QoL. Number of hours worked (*p* = 0.011), being a nursing technician (*p* = 0.041), number of jobs (*p* = 0.042) and quality of life (*p* < 0.001) exerted a significant influence on burnout.

**Table 3:** Generalized linear models and their β coefficients for quality of life and burnout. Botucatu, SP, Brazil, 2022

	Burnout			Quality of Life		
Explanatory variables	β coefficient	Standard error	P	β coefficient	Standard error	p
Age	− 0.01	0.01	0.26	−0.00	0.00	0.02
Hours worked	0.01	0.00	0.01	−0.00	0.00	0.08
Sex						
*Ref.*^a^ Male	−0.30	0.22	0.17	−0.26	0.12	0.03
Occupation						
*Ref.* Nursing technician	0.24	0.12	0.04	−0.19	0.29	0.50
Number of jobs						
2 vs. 1	−0.62	0.30	0.04	−0.56	0.45	0.21
Monthly family income						
*Ref.* R$ 1001 to 3000	2.06	1.37	0.13	0.82	0.31	0.01
*Ref.* R$ 3001 to 5000	2.43	1.40	0.08	0.94	0.31	0.00
*Ref.* More than R$ 5001	2.23	1.41	0.11	1.06	0.32	< 0.00
QoL Total	−0.38	0.07	< 0.00			
Burnout Total				−0.74	0.12	< 0.00

^a^Ref. = reference

## Discussion

The assessment of quality of life and burnout of health professionals have been used to guide interventions and to develop public health policies, especially during the COVID-19 pandemic in several countries [33, 34].

The predominant characteristics of the study sample were being a woman, having a complete higher education, being a nursing technician and not having children. These results are compatible with data described in previous studies conducted in Brazil with nursing staff who work at urgent and critical care units [35, 36]. Most participants earned between R$1.000 to 3.000 (USD 184 to 552) a month, which is lower than that identified in a study conducted in the state of Minas Gerais involving 160 nursing staff, in which 66% of nursing technicians earned R$4000 (USD 736) or more per month [37].

The most affected aspects of QoL were the physical and social relationships domains. These findings may be attributed to the absence of recreational/leisure activities, the impact on financial resources and other factors resulting from the changes imposed by the COVID-19 pandemic [38].

Regarding burnout, the results demonstrated that the nursing staff evaluated had high scores for both the exhaustion and disengagement domains, which, according to the authors of the instrument, constitute burnout syndrome [29]. These results may be attributed to the complexity of patients admitted to ICUs, which can contribute to emotional exhaustion as well as high levels of depersonalization and dissatisfaction with work among the healthcare providers who work in these settings [39].

A study conducted in Iran with 245 participants reported similar findings, identifying greater frequencies of burnout among nurses who have worked on the frontline of the pandemic compared to other nurses [40]. These healthcare providers face several problems, such as anxiety, the fear of infection, excessive work burden, extreme weariness and depression, due to the fact that they work with critically ill patients on a daily basis, which may indirectly contribute to the occurrence of burnout syndrome [41].

A comparative study involving healthcare providers at ICUs before and during the pandemic demonstrated an increase of 23 to 36% in the prevalence of burnout, indicating that the burden of responsibilities and the performance of such services for a prolonged period of time contribute to the development of this syndrome [42].

To date, there are various instruments to assess burnout of health professionals. They aim not only to avoid the adverse consequences of burnout for the personal life of health professionals but also to improve patient care and stimulate the development of preventive strategies at health services [43]. In this context, a new multidimensional instrument called the Burnout Assessment Tool (BAT) was developed to measure six dimensions that capture exhaustion symptoms related to burnout: exhaustion, mental detachment, cognitive impairment, emotional impairment, psychological stress and psychosomatic complaints [43].

However, a study conducted during the pandemic showed the importance of promoting compassionate relationships at the working environment as a preventive strategy to reduce occupational risks in health services. Based on the protective effect of compassion, the authors believed that their study could contribute to the improvement of research instruments used to assess work related stress, since compassion is poorly assessed [44]. Therefore, assessing compassion could improve both the health of professionals and general wellbeing at work [44].

To answer the second research question of the present study, a multiple linear regression analysis was performed with QoL and explanatory variables. This outcome was positively associated with income and negatively associated with age, the male sex and burnout. The negative impact of burnout on QoL may be attributed to the considerable psychological pressure and excessive work burden to which nursing staff in critical care units were exposed during the pandemic. A study conducted in Turkey with 240 healthcare providers found high levels of stress, anxiety and burnout, which exerted a negative impact on QoL [45].

Another important finding was the positive association between income and QoL, although there is no regulation in the country regarding a minimum wage for the profession. Job dissatisfaction potentially generates physical and psychological problems that tend to exert a negative impact on QoL [46]. This situation was also reported in a study conducted at an adult ICU, in which a low monthly income exerted a negative impact on the QoL of nursing staff [47].

In the present investigation, male nursing staff had a poorer QoL. This finding may be due to the fact that the majority of participants were women and, therefore, may have interfered with the statistical analysis.

The results showed a positive association between the occupation of nursing technician and burnout syndrome. A similar finding was reported in a study conducted at intensive and semi-intensive care units at a hospital in São Paulo, in which 12% of the nursing staff had evidence of burnout and 66.67% of these individuals were nursing technicians, which indicates that the syndrome predominantly affects this job description [48]. However, levels of burnout increased significantly with the emergence of the pandemic due to factors linked to the profession, such as an excessive workload, as well as aspects linked to the pandemic itself, such as the fear of infection by the disease [49]. Although all healthcare providers are susceptible to burnout syndrome, the literature reports that nursing staff were the most affected during the pandemic and also the most affected by symptoms of anxiety, fear and depression [50].

In the present study, burnout was positively associated with the number of working hours and negatively associated with the number of jobs. The increase in the number of hours during the pandemic possibly contributed to this result, with an impact on the emotional exhaustion dimension. In contrast, the negative association with the number of jobs was because only 10% of the participants worked in two jobs, which influenced the comparison of this variable.

The present study has limitations that should be considered. The cross-sectional design restricted the interviews to a single moment in time, which may not have been sufficient to portray the magnitude of the changes imposed by the pandemic. The considerable predominance of women in the sample (92.6%) could also be considered a limitation of this study. Thus, further studies should be developed with a longitudinal design to contribute to improvements in the working conditions of nursing staff.

### Contributions of the study

This study highlights the need for healthcare institutions to be prepared for pandemics and possible future outbreaks of COVID-19 with measures of psychological/social support for the protection and follow-up of nursing staff who work on the frontlines of such public health problems. Moreover, better working conditions should be provided so that healthcare providers can obtain adequate rest and replenishment between work shifts.

## Conclusion

The results of the present study indicate that the participants had good QoL scores, although the physical domain was the most compromised. Another data presented is that participants who had symptoms of burnout syndrome had a negative impact on quality of life.

In the analysis of sociodemographic variables, male participants and those with burnout syndrome had a worse perception of this construct, while higher income was considered determinant for improving quality of life. The number of hours worked, and the occupation of a nursing technician were associated with an increase in the incidence of burnout.

At present, the COVID-19 pandemic seems to be under control in Brazil, despite the new variants of the virus. However, it is important that the Brazilian government, health services, and policy makers reorganize and improve the health services so that they are better prepared to manage future pandemics, especially for intensive care units and emergency departments. Ideally, strong mental health support should be provided to health professionals that includes psychological treatment. Future research should investigate whether policies have been implemented to reduce burnout levels of professionals in hospitals. Ultimately, health professionals worldwide, who were at the frontline of the COVID-19 pandemic, deserve better working conditions and recognition for their sacrifice and hard work.

## Data Availability

The datasets generated and/or analysed during the current study are not publicly available to preserve anonymity of the respondents but are available from the corresponding author on reasonable request.
